# Supplementary material for: Modelling Skylarks (Alauda arvensis) to Predict Impacts of Changes in Land Management and Policy: Development and Testing of an Agent-Based Model
Source: PLoS One. 2013 Jun 6;8(6):e65803. doi: 10.1371/journal.pone.0065803 (PMC3675089; doi:10.1371/journal.pone.0065803)
Supplement: Supporting Information S4 — The skylark ODdox as a zipped archive. (ZIP) [file pone.0065803.s004.zip › Skylark_ODdox/farmfuncs_8cpp.html]

ALMaSS Skylark ODdox: farmfuncs.cpp File Reference


ALMaSS Skylark ODdox
 2.0


- Main Page
- Related Pages
- Classes
- Files

- File List
- File Members

Macros |
Variables

farmfuncs.cpp File Reference

**Farmfuncs.cpp This file contains the source for implementing the farm events**   
More...

`#include "ls.h"`

|  |  |
| --- | --- |
| Macros | |
| #define | DO\_IT\_PROB   (l\_farm\_event\_do\_it\_prob.value()) |
| #define | UNREFERENCED\_PARAMETER(P)   (P) |

|  |  |
| --- | --- |
| Variables | |
| Landscape \* | g\_landscape\_p |
| static CfgFloat | l\_farm\_cattle\_veg\_reduce ("FARM\_CATTLE\_VEG\_REDUCE", CFG\_PRIVATE, 0.10) |
| static CfgFloat | l\_farm\_cattle\_veg\_reduce2 ("FARM\_CATTLE\_VEG\_REDUCE\_LOW", CFG\_PRIVATE, 0.025) |
| static CfgInt | l\_farm\_event\_do\_it\_prob ("FARM\_EVENT\_DO\_IT\_PROB", CFG\_PRIVATE, 50) |
| static CfgBool | l\_farm\_herbicide\_kills ("FARM\_PESTICIDE\_KILLS", CFG\_CUSTOM, true) |
| static CfgBool | l\_farm\_insecticide\_kills ("FARM\_INSECTICIDE\_KILLS", CFG\_CUSTOM, true) |
| static CfgFloat | l\_farm\_pig\_veg\_reduce ("FARM\_PIG\_VEG\_REDUCE", CFG\_PRIVATE, 0.98) |
| CfgFloat | l\_pest\_insecticide\_amount |
| CfgFloat | l\_pest\_product\_0\_amount |
| CfgFloat | l\_pest\_product\_1\_amount |
| CfgInt | l\_pest\_productapplic\_enddate |
| CfgInt | l\_pest\_productapplic\_period |
| CfgInt | l\_pest\_productapplic\_startdate |

---

## Detailed Description

**Farmfuncs.cpp This file contains the source for implementing the farm events**

by Frank Nikolaisen & Chris J. Topping   
Initial version of June 2003, but under continual change.   
All rights reserved.   
  
Doxygen formatted comments in July 2008

---

## Macro Definition Documentation

#define DO\_IT\_PROB   (l\_farm\_event\_do\_it\_prob.value())

Referenced by Farm::AutumnHarrow(), Farm::AutumnPlough(), Farm::AutumnRoll(), Farm::AutumnSow(), Farm::BurnStrawStubble(), Farm::CattleOut(), Farm::CattleOutLowGrazing(), Farm::CutToHay(), Farm::CutToSilage(), Farm::CutWeeds(), Farm::DeepPlough(), Farm::FA\_AmmoniumSulphate(), Farm::FA\_GreenManure(), Farm::FA\_Manure(), Farm::FA\_NPK(), Farm::FA\_PK(), Farm::FA\_Sludge(), Farm::FA\_Slurry(), Farm::FP\_GreenManure(), Farm::FP\_LiquidNH3(), Farm::FP\_ManganeseSulphate(), Farm::FP\_Manure(), Farm::FP\_NPK(), Farm::FP\_NPKS(), Farm::FP\_PK(), Farm::FP\_Sludge(), Farm::FP\_Slurry(), Farm::FungicideTreat(), Farm::GrowthRegulator(), Farm::Harvest(), Farm::HayBailing(), Farm::HayTurning(), Farm::HerbicideTreat(), Farm::HillingUp(), Farm::InsecticideTreat(), Farm::Molluscicide(), Farm::PigsOut(), Farm::RowCultivation(), Farm::SpringHarrow(), Farm::SpringPlough(), Farm::SpringRoll(), Farm::SpringSow(), Farm::StrawChopping(), Farm::Strigling(), Farm::StriglingSow(), Farm::StubbleHarrowing(), Farm::Swathing(), Farm::Water(), and Farm::WinterPlough().

|  |  |  |  |  |  |
| --- | --- | --- | --- | --- | --- |
| #define UNREFERENCED\_PARAMETER | ( |  | P | ) | (P) |

---

## Variable Documentation

Landscape\* g\_landscape\_p

CfgFloat l\_farm\_cattle\_veg\_reduce("FARM\_CATTLE\_VEG\_REDUCE", CFG\_PRIVATE, 0.10)

static

Referenced by Farm::CattleIsOut(), and Farm::CattleOut().

CfgFloat l\_farm\_cattle\_veg\_reduce2("FARM\_CATTLE\_VEG\_REDUCE\_LOW", CFG\_PRIVATE, 0.025)

static

Referenced by Farm::CattleIsOutLow(), and Farm::CattleOutLowGrazing().

CfgInt l\_farm\_event\_do\_it\_prob("FARM\_EVENT\_DO\_IT\_PROB", CFG\_PRIVATE, 50)

static

CfgBool l\_farm\_herbicide\_kills("FARM\_PESTICIDE\_KILLS", CFG\_CUSTOM, true)

static

Referenced by Farm::HerbicideTreat().

CfgBool l\_farm\_insecticide\_kills("FARM\_INSECTICIDE\_KILLS", CFG\_CUSTOM, true)

static

Referenced by Farm::InsecticideTreat(), Farm::ProductApplication0(), Farm::ProductApplication1(), and Farm::SynInsecticideTreat().

CfgFloat l\_farm\_pig\_veg\_reduce("FARM\_PIG\_VEG\_REDUCE", CFG\_PRIVATE, 0.98)

static

Referenced by Farm::PigsAreOutForced(), and Farm::PigsOut().

CfgFloat l\_pest\_insecticide\_amount

CfgFloat l\_pest\_product\_0\_amount

Referenced by Farm::ProductApplication0().

CfgFloat l\_pest\_product\_1\_amount

Referenced by Farm::ProductApplication1().

CfgInt l\_pest\_productapplic\_enddate

CfgInt l\_pest\_productapplic\_period

CfgInt l\_pest\_productapplic\_startdate


- CJT
- MSVC
- ALMaSS Working Source
- Landscape
- farmfuncs.cpp
- Generated on Thu Jan 10 2013 13:15:35 for ALMaSS Skylark ODdox by
   1.8.1.1
